# Exploring Fracture Patterns: Assessing Representation Methods for Bone Fracture Simulation

**DOI:** 10.3390/jpm14040376

**Published:** 2024-03-30

**Authors:** Francisco Daniel Pérez-Cano, Gema Parra-Cabrera, Ivett Vilchis-Torres, José Javier Reyes-Lagos, Juan José Jiménez-Delgado

**Affiliations:** 1 Department of Computer Science, University of Jaén, 23071 Jaén, Spain; gparras@ujaen.es (G.P.-C.); juanjo@ujaen.es (J.J.J.-D.); 2 Centro de Investigación Multidisciplinaria en Educación, Universidad Autónoma del Estado de México, Toluca 50110, Mexico; ivilchist@uaemex.mx; 3 Facultad de Medicina, Universidad Autónoma del Estado de México, Toluca 50110, Mexico; jjreyesl@uaemex.mx

**Keywords:** bone fracture, fracture pattern, medical simulation, segmentation, image analysis

## Abstract

Fracture pattern acquisition and representation in human bones play a crucial role in medical simulation, diagnostics, and treatment planning. This article presents a comprehensive review of methodologies employed in acquiring and representing bone fracture patterns. Several techniques, including segmentation algorithms, curvature analysis, and deep learning-based approaches, are reviewed to determine their effectiveness in accurately identifying fracture zones. Additionally, diverse methods for representing fracture patterns are evaluated. The challenges inherent in detecting accurate fracture zones from medical images, the complexities arising from multifragmentary fractures, and the need to automate fracture reduction processes are elucidated. A detailed analysis of the suitability of each representation method for specific medical applications, such as simulation systems, surgical interventions, and educational purposes, is provided. The study explores insights from a broad spectrum of research articles, encompassing diverse methodologies and perspectives. This review elucidates potential directions for future research and contributes to advancements in comprehending the acquisition and representation of fracture patterns in human bone.

## 1. Introduction

The accurate identification and extraction of fracture patterns in human bones has a key role in the improvement of medical care and the development of advanced medical technologies. The ability to discern and analyze specific bone fracture patterns is essential for healthcare professionals, as it provides crucial information for diagnosis, treatment, and planning of surgical interventions. Furthermore, the automated extraction of these patterns through innovative techniques, such as the use of deep learning algorithms, not only streamlines the process, but also contributes to standardization and accuracy in the interpretation of images by medical trauma professionals.

The population in many countries is aging, so fracture studies are becoming even more relevant. As the population ages, there is a significant increase in cases of bone fractures related to the wear and tear and bone fragility associated with aging [1]. The ability to analyze these fracture patterns in detail becomes essential to better understand the clinical implications and establish good strategies for treatment. A rapid and accurate response to needs contributes directly to the recovery process of patients. At advanced ages, one of the most common cases of fracture is hip fracture, which has a mortality rate of between 5% and 10% in the first month and multiplies over the recovery period [2].

The purpose of this work is to carry out a review of the relevant literature that addresses the process of acquiring fracture patterns in bones. Moreover, the article aims to examine the different methodologies used to represent these fracture patterns, evaluating their effectiveness and accuracy. Finally, the goal is to summarize existing knowledge, identify potential gaps in advancing this line of research, and provide a comprehensive perspective that contributes to the progress in understanding the acquisition and representation of fracture patterns in human bones. To this end, a wide variety of research articles are reviewed in this work.

The introduction sets the stage by outlining the significance of acquiring and representing fracture patterns in human bones for various medical applications. Following this, the article delves into the methodologies involved in pattern acquisition, exploring techniques such as segmentation algorithms and curvature analysis. Subsequently, the focus shifts to pattern representation methods, including texture-based, point cloud, and spherical coordinate representations. The discussion section critically analyzes the effectiveness and suitability of each method for specific medical applications, shedding light on challenges and potential advancements. Finally, the article concludes by synthesizing key findings, identifying gaps for future research, and proposing avenues for further exploration in understanding fracture pattern acquisition and representation.

## 2. Pattern Acquisition

The process of obtaining fracture patterns involves a comprehensive approach based on image analysis. To this end, the initial step entails the precise capture of images to identify and delineate the area affected by the fracture. Subsequently, advanced image processing techniques are employed to extract specific features from the fractured area, including the arrangement, extent, and number of fracture lines that make up the fracture.

The shape of bones presents a great challenge given the roughness and anatomical features. Each human bone is unique and has geometry and dimensions that are different from the bones in other specimens [3,4]. Factors such as nutrition, age, and lifestyle influence the shape and size of the bones; thus, it is important to use advanced methodologies in order to analyze the characteristics of each fracture [5]. Despite this, they share a similar structure and weaknesses in the same regions of the bone. Therefore, the main classifications of bone fractures are based on the region in which they occur and the number of fragments generated rather than the exact shape of the fractures [6,7]. Moreover, Perez et al. [8] recently studied the repeatability of a fracture pattern in human femurs within a controlled environment and concluded that, although the characteristics are unique, they share fragility in the same areas and the conditions can be reproduced to simulate the same type of fracture.

In the medical field, computed tomography (CT) images are often used to represent the bone structures in the human body. These images represent multiple cross-sectional images, or slices, taken by a CT scan. These images are usually combined by a computer to produce detailed 3D representations of organs, tissues, and bones. In order to obtain such detailed representations, image segmentation and analysis algorithms are often used. These representations are the most accurate in order to analyze a bone fracture pattern.

### 2.1. Bone Model Segmentation

In order to obtain an accurate representation of the human bone fracture pattern, the first step is to segment the CT images of the bones. Traditional methods work well with healthy bones but are unable to identify fractured bones. In addition, most studies focus on the segmentation of a particular fracture case, so there is a wide range of work on bone segmentation. However, previous work has been developed based mainly on three approaches: threshold-based methods, region-growing-based methods, and deep learning.

Neubauer et al. [9] extracted the radius, ulna, and carpus from the CT scan to generate three masks and filter the bone from other tissues using a threshold defined by the user. Then, they used a watershed-based algorithm to separate erroneously joined fragments in a fracture case. Some manual corrections need to be performed in case of inaccuracies. Shadid and Willis proposed a probabilistic version of the watershed algorithm to expand regions along constant contours of likelihood [10]. Tomazevic et al. [11] also used a threshold-based method with the manual intervention of the user. In [12], the authors implemented a multi-threshold combined with the K-Means algorithm to segment the cortical bone. The CT images were clustered into foreground and unwanted regions. The models were then segmented following the traditional technique, and the information of the unwanted regions was eliminated.

Several authors use a region-growing-based method [13,14,15]. They combine the use of an adaptive thresholding method with a 2D region growing. To separate the bones that make up the bone fracture, they apply a 3D connected component-labeling algorithm. Fornaro et al. [13] used an interactive graph-cut method to improve weak edges and to separate wrongly connected bone pieces. Subsequently, some authors began to propose methods based on the use of multiple seeds to avoid problems when segmenting the different fragments that make up a fracture [16,17]. In [17], the authors proposed a method consisting of a semi-automatic procedure to segment a CT scan, in which the user manually places seeds to separate fragments with touching zones. After placing the seeds, these seeds are propagated through the image pile, discarding those regions containing a certain level of noise. A curvature flow filter is also applied to each slice before the segmentation process to smooth the images. This algorithm also resolves certain special cases where overgrowth occurs in the regions. Ruikar et al. [18] added a preprocessing step, in which they used the histograms of the images to remove the flesh surrounding the bones during segmentation and erase the various artifacts that occur, thus improving the results obtained after seed expansion.

The program 3DSlicer [19] is an image-computing platform that contains a collection of tools widely used in medical image processing, that allows users to segment different tissues from CT scans. This platform contains all the traditional methods mentioned above. The users can determine which method is more appropriate, depending on the characteristics of the clinical case. Simpleware Software, Invivo5, Romexis, Materialise Mimics, and OsiriX [20] are other platforms that allow segmentation of bone models through medical imaging. However, 3D Slicer is better for dimensional and geometric accuracy in 3D reconstruction for bone models. Mandolini et al. [21] analyzed the accuracy of the segmentations obtained with different platforms and concluded that 3D slicer, in addition to the advantage of being open source, was the software that offered the lowest average deviation when reconstructing human femurs.

In 2017, a new trend began, in which some authors started to use deep learning-based techniques for bone segmentation [22,23,24,25,26,27,28,29,30,31,32,33]. The main problem with these works is that they focus on a specific part of the human body and are not generic models. In [27], authors presented a 3D feature network to achieve fast and accurate femur segmentation. They used two components, including the edge detection task and the multiscale feature fusion segmentation task, to train the model and improve the quality of the results. Some authors started to develop a model for the segmentation of whole bones [22,29]. Klen et al. [22] proposed a model using three different strategies: training from 2D axial slices, a pseudo-3D approach (including axial, sagittal, and coronal slices), and an approach where the network is pre-trained in an unsupervised manner. The accuracy of the model obtained compared to the ground truth was very high with all three strategies. They used different metrics to evaluate the results by comparing the generated model with the ground truth created by an expert. Noguchi et al. [29] focused on whole bones by using a U-Net architecture. They also evaluated the efficacy of three types of data augmentation techniques. The most recent works using this approach and the parts of the body for which they were developed are compiled in [34].

Table 1 summarizes the characteristics, advantages, and disadvantages of each method for the segmentation of human bones.

### 2.2. Fracture Zone Analysis

Once the model is obtained, the 3D bone models are processed to locate candidate points for fractured area identification. Detecting the precise area of a fracture from medical images poses significant challenges. One primary difficulty lies in obtaining accurate segmentation of bone fragments, particularly in multifragmentary fractures. These fractures often involve small, irregularly shaped bone fragments that can be challenging to differentiate from surrounding tissue. Moreover, the need for precise identification and separation of these fragments adds complexity to the segmentation process, requiring sophisticated image-processing algorithms, such as curvature or normal filters, and careful manual intervention.

Another challenge arises in cases where there is no clear plane facing the fracture zone. In such instances, additional time and effort may be required for clinicians or researchers to meticulously analyze the images and ensure a successful fracture reduction. This increased user interaction time not only prolongs the diagnostic process but also adds to the complexity of treatment planning.

Complex fractures further compound the difficulties in accurately identifying and characterizing the fracture zone. Fractures with multiple fragments or deformations present unique challenges, as the fractured area may exhibit irregular shapes and orientations. Distinguishing between different fragments and accurately delineating the boundaries of the fracture zone become intricate tasks, requiring advanced imaging techniques and expert interpretation.

In order to solve these drawbacks, several approaches can be found in the previous literature. Winkelbach et al. [35] took advantage of the specific shape of cylindrical bones. In order to identify vertices of the fractured area, they checked the normal orientation of each vertex and compared it with the bone axis. This method does not work when fracture lines are almost parallel to the bone axis.

Other authors have proposed interactive methods to identify fracture surfaces in craniofacial fractures [36]. In these works, fracture contours are extracted interactively from segmented bone fragments. With that aim, specialists have to select points belonging to the fractured area, and then a contour-tracing algorithm generates the remaining points.

Some statistical approaches have been proposed to identify fractured zones. Willis et al. [37] used a mixture model consisting of two Gaussian probability distributions to perform a binary classification that allowed separation of intact and fractured zones in each bone fragment. After classifying all points, the fractured surface is the largest continuous region of fractured surface points. Zhou et al. [38] presented an extension of the previous method. They added a human-assisted stage in which they refined the selected fracture zone to improve the fragment alignment in bone fracture reduction simulation.

Curvature analysis has also been used to identify fractured surfaces. Curvature filters are used to analyze the smoothness and curvature changes in the surface represented by the point cloud. Okada et al. [39] presented a curvature-based procedure to obtain fracture lines in each slice. For that purpose, they generate a 3D curvature image from the CT stack. Then, interactive line-tracking software allows extraction of the fracture lines from the generated 3D curvature image. Kronman and Joskowicz [40] identified fracture surfaces using intensity and curvature filters. Then, outliers were removed from the estimated contact surface with a connectivity test. Buschbaum et al. [41] also identified fracture lines using surface curvatures. The strongly curved edges were selected and fracture lines were generated.

Fürnstahl et al. [42] used a normal-based filter to identify candidate points that may belong to the fracture surface. Then, connected component analysis was applied in order to remove outliers.

Paulano and Jiménez [43] developed a method to identify the fracture zone from a fractured bone in order to reduce a fracture. This method uses an oriented bounding box (OBB) to enclose the model, and it is discretized using a grid, using the direction of the OBB defined by the vector that joins the centroids of every pair of fractured models to perform the fracture reduction. Afterwards, the grid is traversed through the parallel columns to the sweep vector and the points that belong to the first populated voxel in each column are marked as candidate points. Finally, the candidate points are filtered based on their distance to the candidate points of the opposite fragment. Luque-Luque et al. [44] proposed an improvement to this method by using an oriented plane to obtain a refined fracture zone. They also used filters based on curvature and statistical parameters to remove outliers. In subsequent work, Luque-Luque et al. [45] added an additional filter using seeds to avoid, or reduce, the manual cleaning of the bone fracture surface. These seed points serve as initial reference points for the fracture zone identification process. They are then expanded to encompass all points within the fracture zone, utilizing region-filtering techniques to clean outliers or isolated areas that are not part of the fracture zone. In each iteration, the neighborhood of the initial set is checked using curvature and normal filters that determine the candidates to be considered as part of the fracture in the next iteration. In both algorithms, the cortical area identification is performed with a projection of the point cloud on the fitting plane calculated previously.

Recently, an algorithm for the extraction of potential fracture zones of bones has been presented by Zeng et al. [46]. They introduced a density-based clustering algorithm to automatically detect the fracture zone points with higher density. The algorithm defines areas with a sufficient point cloud density in the neighborhood as clusters and other areas as noise. The problem with this approach is that the fracture zones may contain a high level of noise. The deformations of the bone itself can lead to areas of the model with a high density of geometry that can be identified as fracture zones.

Some authors also used deep learning techniques for the automated fracture detection from medical images [47,48,49,50,51]. However, they can only classify or identify the area where it is produced, so this information cannot be used in a later simulation with human bones.

Although there are several alternatives when it comes to filtering fracture zone information, many of the studies combine the use of several methods and filters to refine the result. The main methods that have been found in the literature, along with their advantages and disadvantages, can be found in Table 2.

## 3. Pattern Representation

A fracture pattern is an abstract model that represents the fractured area of a bone. These fracture patterns can be used to replicate a fracture in another area of the same bone or in a different bone with similar features.

The adequate representation of a fracture pattern is necessary because it provides a high degree of accuracy in the determination of the fracture zone, as well as being necessary to compare fracture patterns and to catalogue them according to a set of criteria.

There are several ways to represent a fracture pattern. The most common methods are by textures, point clouds, and spherical coordinates. The following sections show the way in which the information is represented and how it is obtained with all the methods presented above. Thus, the best approach to represent the fracture zone can be identified to perform fracturing simulations.

### 3.1. Textures

**Figure 1:**
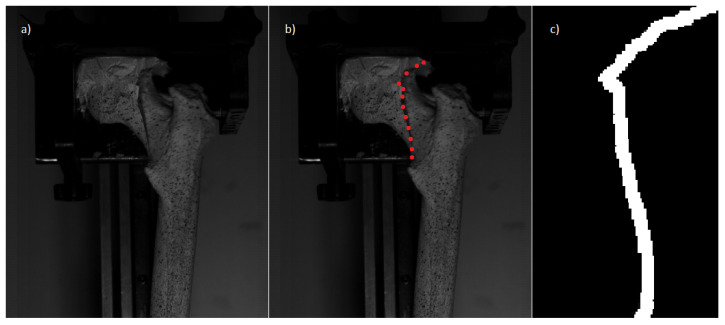
Manual selection of the fracture zone performed by Pérez-Cano et al. [55]. Figure (**a**) represents the exact moment of the bone fracture. Figure (**b**) the points that make up the fracture line drawn by the user and Figure (**c**) shows the bone fracture pattern obtained.

Fracture patterns can be represented with 2D images. These are the easiest and simplest representations. They are also the most inaccurate. Use of 2D images is one of the most widely used representations in the previous literature on geometric fracturing [52,53,54,55]. In bone fracturing, this representation does not require the use of CT images to obtain the fracture pattern; it requires an analysis of the fracture zone of real bone specimens, utilizing a variety of imaging modalities, including X-rays, photographs, and sequential images where the exact moment at which the fracture occurs is identified. In order to obtain these, an analysis of the fracture zone of the real bone is performed. Therefore, it is necessary to monitor the exact moment when a fracture occurs. This is because the displacement can cause small pieces to break off, altering the original position at which the fracture occurred. The capture and analysis of the fracture lines in a posterior way may result in the bone arrangement not being exactly the same in all cases of study. Figure 1 shows an overview of the process to be followed to obtain the fracture pattern. To obtain the fracture lines, the frame where the fracture occurs is identified (Figure 1a) and the entire area is marked manually (Figure 1b,c).

**Figure 2:**
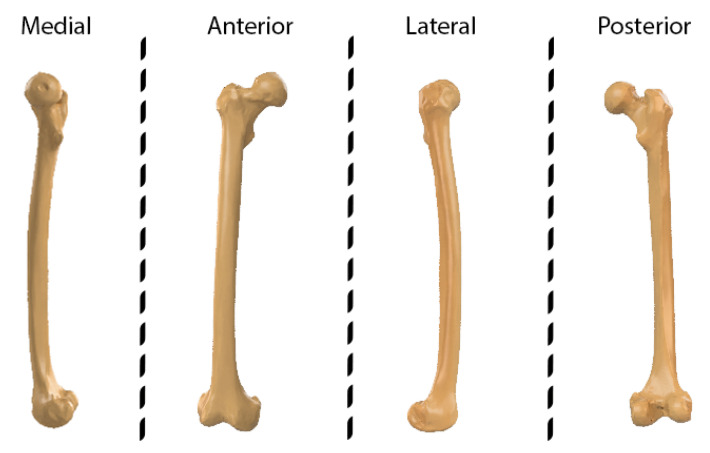
Representation of the four aspects in a femur.

In these patterns, the fracture lines are represented with two colors. One color represents the zones where there is no fracture line, while the other represents the fracture line. Each image represents the fracture line from one of the views of the geometric model. Figure 2 shows the main aspects of the long bones: anterior, posterior, lateral, and medial. As this representation captures only the information from a specific aspect of the bone, it inherently imposes limitations on the scope of simulations that can be conducted using this fracture pattern. A more ambitious approach could combine the patterns obtained from the different aspects of the bone to obtain a 3D representation of the fracture. However, these patterns are used because of their simplicity. Studies that seek a more faithful representation of the fractured zone are based on the use of segmented models through CT images. These works allow one to obtain the fractured zone with a high level of detail, as is shown in the following sections.

### 3.2. Point Clouds

**Figure 3:**
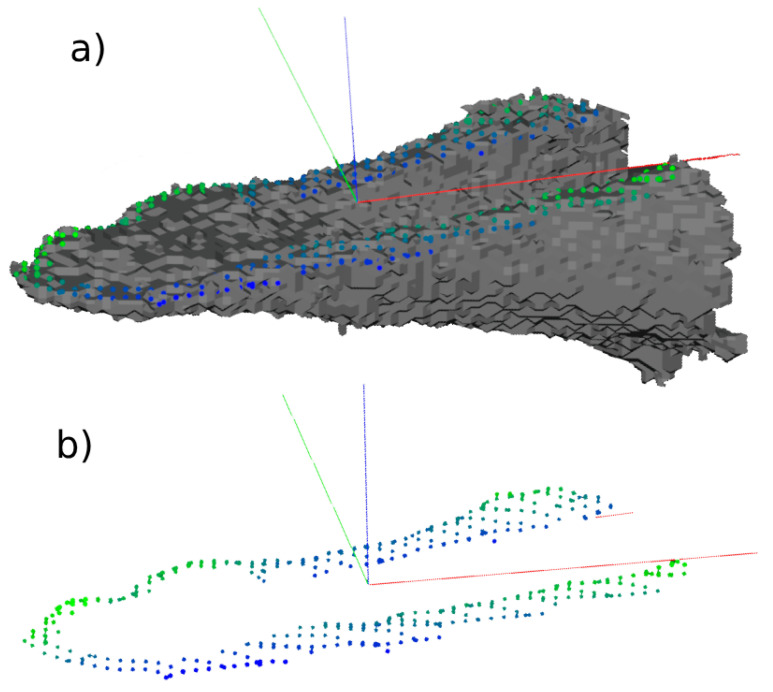
Clinical case of a humerus fracture. Figure (**a**) shows the points of the fracture area overlapped on the bone fragment while (**b**) shows the points of the area of the fracture after using some filters. The color of the dots represents the difference in height between the dots. The green-colored dots represent the higher ones, while the blue ones represent the lower ones.

A point cloud is a set of points that represent and define the geometry of a 3D object. In bone simulations, point clouds can be used to represent the bone models and the fracture zone. A set of points in a 3D space allows one to faithfully represent the position of the points of the fracture zone, allowing one to know perfectly the dimensions of the fracture zone and its location (Figure 3). A fracture pattern can be obtained from the fracture zone defined by a point cloud. This is the most-used representation of bone fracture patterns since most of the work in this area focuses on the fracture-reduction process, which involves 3D representations of the bone fractures [35,36,37,43,44,45,46].

**Figure 4:**
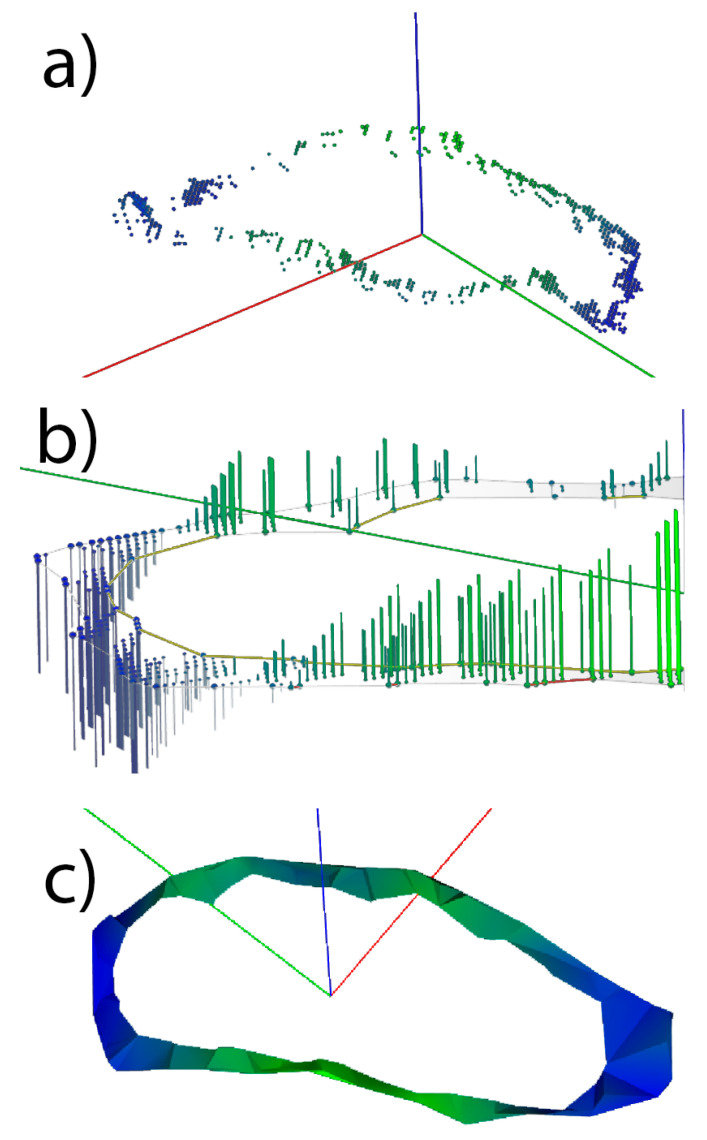
Different methods of representing a pattern starting from a point cloud. Figure (**a**) shows the point cloud extracted from the clinical case while Figure (**b**) shows these points projected to a plane using the color to define the height of the points. Figure (**c**) present a triangle mesh which join the points that make up the fracture.

There are several methods to represent the fracture pattern obtained such as the point cloud itself, height maps, or triangle meshes. The representation of a fracture zone by a point cloud or its variants offers substantial utility in the detailed characterization of fractures in materials. In Figure 4a, we have the points in their original position with the associated color; meanwhile, in Figure 4b, they are projected on a 2D plane, including the border that delimits the fracture zone and a projection of the points on the plane representing the distance to it. These points can be processed by a 2D alpha shape algorithm that returns the cortical surface of the pattern (Figure 4c). The points are in a range of colors from blue to green, in which blue indicates the points farthest from the fitting plane while green indicates the points closest to the fitting plane.

The point cloud provides a faithful representation of the fractured surface, while the height map and the triangle mesh provides a more organized structure for further analysis. The use of a height map provides additional information on topology and vertical variation in the fracture. A representation using a triangle mesh accurately captures the fracture topology and geometry, allowing for a more complete and detailed visualization. This three-dimensional representation not only facilitates visual inspection of the fracture, but also provides the opportunity for quantitative analyses, such as crack orientation measurement and fracture surface roughness evaluation. In addition, the mesh representation facilitates the integration of data into simulation models and structural analysis, which can be valuable in predicting the mechanical behavior of materials in the presence of fractures.

### 3.3. Contour Plot

**Figure 5:**
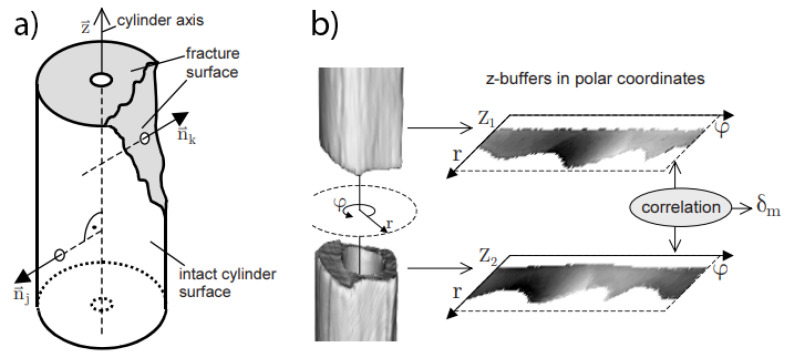
Representation of the fracture pattern by contour plots. (**a**) shows how the normals are compared to identify the fracture zone, while (**b**) shows how this information is projected onto a plane to obtain the fracture pattern representation.

The contour plot is a bone fracture pattern representation proposed by Winkelbach et al. [35]. They took advantage of the cylindrical shape of bones to detect the fractured area. In order to identify fracture patterns, they built an orthographic Z-buffer for each fragment and aligned them to find the correct rotation of the fragments. The procedure used for the fracture representation consisted of two stages. The first one selected the axis for the cylinder through the Hough transformation. The axis of the cylinder relied on the fact that all normals to the surface of the cylinder are perpendicular to its axis (Figure 5a). The second stage consisted of comparing the normals to the bone axis (Figure 5b). Vertices that were neither collinear with the radius of the cylinder nor perpendicular to the axis were marked as vertices belonging to the fracture zone. This method only applies to cylindrical bones and does not work when fracture lines are almost parallel to the bone axis or if the fracture is not in the diaphysis area. These patterns can be projected onto a 2D plane, resulting in a two dimensional pattern with 3D fracture information.

### 3.4. Spherical Coordinates

**Figure 6:**
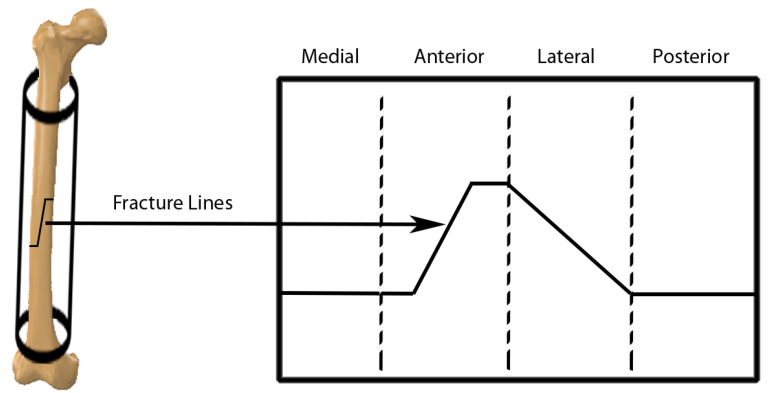
Scheme for the transformation of spherical coordinates to a 2D representation.

Spherical coordinate systems are based on the same idea as polar coordinates and allow us to represent a position in space. These coordinates represent positions in space in problems that unfold over a cylindrical shape. For this representation, three coordinates are used, with one of them being the angle with the *X* axis of the cylinder and the remaining two being distances. The use of spherical coordinates to present a fracture pattern can be simplified by projecting them onto a 2D plane, resulting in a two-dimensional pattern with 3D fracture information. This process involves mapping each point on the bone surface to its corresponding coordinates in a spherical coordinate system, where the radial distance, polar angle, and azimuthal angle uniquely define each point. Subsequently, these spherical coordinates are transformed into Cartesian coordinates, allowing for the projection of the fracture pattern onto a 2D plane. By unfolding the fracture zone in this manner, the resulting representation provides a detailed and comprehensive view of the fracture characteristics, facilitating in-depth analysis and classification of fracture patterns for various medical applications. Figure 6 shows an example of the projection in a plane. Several authors used this representation to analyze bone fracture patterns, subdividing the spatial plane in which the fracture is projected into four parts [56,57,58,59]. Each part corresponds to a view or aspect of the bone (anterior, lateral, posterior, medial).

The aspect on which the 2D representation starts is irrelevant since the order of the views is always the same and the last view connects with the first one when it is transferred to the 3D model. It is a very convenient representation when handling fracture pattern information. Moreover, it allows analysis of the fracture lines with a high level of detail. However, it is not compatible with all bones since, in some cases, the morphology of the bone differs from the cylindrical shape in the epiphyseal or metaphyseal area of the bones.

## 4. Discussion

The previous sections have shown the most relevant works for obtaining a fracture pattern and the different ways to represent them in order to carry out different medical simulations. The importance of using fracture patterns for medical simulation is paramount in the realm of medical research and practice. The accurate representation of fracture patterns, obtained through advanced image analysis techniques, serves as a foundational element for simulations aimed at medical training and diagnostics. Simulations based on realistic fracture patterns provide invaluable insights for orthopedic surgeons.

The acquisition of fracture patterns is not a trivial process. Advanced image analysis techniques are used to accurately identify the fracture zone. In order to obtain a good representation of the fracture pattern, it is ideal to start from a virtual representation of the fractured model in 3D. Direct extraction of fracture patterns from CT images leads to inaccuracies because the quality may vary from one slice to another. In addition, fractures may extend in several directions and affect different parts of the bone so that the CT image stack would have to be examined exhaustively. In traumatology, most work focuses on the use of 3D models reconstructed from CT images as they allow the clinical case to be studied from all possible angles [60]. This helps to better understand the extent and shape of the fracture.

There are numerous studies that focus on obtaining a segmented model. Traditional methodologies work well with healthy bones, but, for fractured cases, it is necessary to use certain modifications of these algorithms. There are two main groups into which we can categorize the techniques: those based on a threshold and those based on region growing. The latest work in the field combines both techniques in order to improve the results obtained. However, despite the large number of studies, most of them are limited to the segmentation of a specific bone and do not work well with any model. The platform developed by Fed [19] can be highlighted for its great versatility and the integration of the different methods. In recent times, several models based on deep learning have emerged that, unlike traditional methods, do not require direct user interaction. In the bone area, the work carried out by Klein et al. [22] stands out due to the high percentage of coincidence with the models reconstructed by experts.

Concerning the analysis of the fracture zone to obtain the fracture pattern and use it in different simulations, the authors have adapted the characteristics of the pattern to the study being carried out. Most of the works related to fracture reduction use the identification of the fracture pattern as a reference to perform the simulation. In some works such as the one developed by Luque-Luque et al. [45], a very precise identification of the fracture zone is used to carry out the simulation. However, other authors use only part of the fracture zone to perform the fracture reduction. Therefore, the accuracy and resources required to obtain a fracture pattern must be linked to the purpose for which they are to be used. The algorithms that allow the zone to be identified most accurately are those in which the methods combine the use of curvature and normal filters. In addition, all processes usually involve a manual cleanup stage to refine the fracture zone. Given the complexity at the geometric level required for fracture zone analysis, there is no fully automatic technique for fracture pattern extraction or deep learning models that currently deals with this problem.

**Figure 7:**
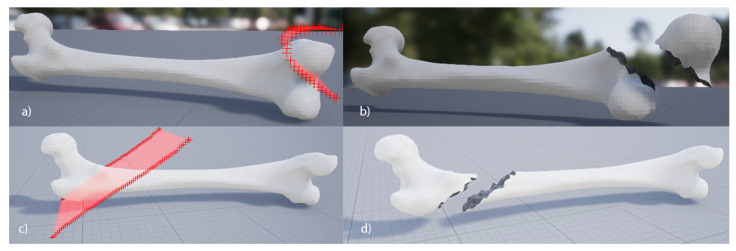
Fracturing simulation using textures performed at [55]. (**a**,**c**) represent the bone prepared to be fractured with the fracture patterns already projected. (**b**,**d**) represent the result obtained after being fractured.

Four alternatives have been found to represent a bone fracture pattern in the previous literature: textures, point clouds, spherical coordinates, and contour plots. Each representation shown above is usually used for a specific purpose; the different variants of point clouds are the most commonly used. The texture-based representation is the simplest and easiest to obtain. It is well-suited for educational purposes and introductory training simulations where simplicity and ease of understanding are paramount. They offer a straightforward visualization of fracture patterns and can be useful for providing basic insights into fracture mechanics. Additionally, they may find application in early-stage fracture diagnosis systems where rapid assessment is needed. Works such as those developed by Müller et al. [61] or Pérez-Cano et al. [55] used this representation. In both studies, they used this representation to combine realistic-looking fracturing with physics-based fracturing. Pérez-Cano et al. [55] applied the texture on the bone model and projected onto one of the aspects of the bone, obtaining the cut-out area (Figure 7a,c). After that, they established a relationship between the different fragments composing the bone fracture and a series of mechanical properties to generate a prefractured model and simulate the detachment of the fragments by applying forces.

The main limitation of this representation lies in the insufficient representativeness of the obtained fracture pattern. This is attributed to the fact that pattern information is available for only one aspect of the bone, lacking comprehensive details about the fracture zone from a 3D perspective. Some medical simulations, such as the automatic reduction of a fracture, require a fairly accurate knowledge of the fracture zone. To address this limitation and enhance the fidelity of the representation, a more comprehensive approach becomes necessary. The other representations have been shown solve this problem. However, these approaches involve capturing the fracture pattern using complex image analysis techniques which require manual processing by the user. As a consequence, the computational cost and time required to obtain this type of representation increase considerably.

For more advanced simulation scenarios and intervention support systems, where high-fidelity and comprehensive fracture information are crucial, point cloud representations are excellent. The large amount of information they provide makes them the most widely used representation for human bone simulations. All simulations in virtual environments use point clouds and triangle meshes to represent bone models. They are ideal for realistic fracture simulations used in surgical planning, patient-specific treatment simulations, and virtual reality-based surgical training systems. Their ability to capture detailed fracture morphology makes them invaluable for accurate fracture reduction planning and execution. Moreover, the information represented using these patterns can be easily extrapolated to other clinical cases. Recently, we found several works such as [45,46,62] in which the authors use this representation as a basis for their studies for bone simulations. The main problem associated with this representation is associated more with the process required to obtain the representation than the representation itself, due to the cost and the need for the user to be directly involved.

The spherical coordinates and contour plot representations are very similar, although they store the information differently. The contour plot representation is an obsolete representation since the amount of information stored in this representation is insufficient for further analysis or other types of simulations. It is the representation on which the spherical coordinate representation was based. This representation shows the fracture zone more faithfully and allows it to be analyzed in great detail. The main line of work that has been developed with this representation of the fracture zone is forensic analysis [56,57,58,59]. This highlights the bone fracture characteristics of each aspect of the bone, making it valuable for forensic experts and clinicians involved in fracture pattern analysis. While less common in clinical settings, it can provide valuable insights into fracture patterns and their implications for forensic investigations. The extended representation in a 2D plane allows the identification of some parameters interesting when classifying a fracture, analyzing its characteristics or planning a treatment. For example, in [59], the authors analyzed the number of fracture lines in each aspect of the bone for all types established by the AO Trauma International and Orthopaedic Trauma Association Representatives (AO/OTA) [7] and showed the relationship between different elements involved in the bone fracture procedure.

Although its strong point is the large amount of information in its representation, the use of a 3D environment can be complex. It is necessary to reverse the initial process for the representation of the pattern. This would require projecting the pattern as if it were a piece of paper wrapped around the bone. In addition, it should be remembered that the pattern starts from a representation of a cylinder shaped area. Therefore, bone ends containing certain deformations should be treated separately. Moreover, it shares the same acquisition problems as the point cloud based representation.

Regarding the applications and uses of the different representations, we can summarize the suitability of the proposed methods. Table 3 shows the main advantages and disadvantages of each representation method. The suitability of each representation method for medical applications depends on factors such as the level of detail required, the complexity of the simulation or analysis task, and the intended audience. Therefore, selecting the most appropriate representation method is crucial for achieving accurate and effective outcomes in medical simulations, intervention support systems, training programs, and educational endeavors.

## 5. Conclusions and Future Work

In this work, an extensive literature review on the acquisition and representation of a bone fracture pattern has been carried out. We started by looking for key terms related to advanced image analysis techniques for fracture zone acquisition in the main scientific article databases. In terms of acquisition techniques, the process involves two main stages: segmentation of the fractured bone and analysis of the fractured zone. Most of the segmentation proposals reviewed are based on very specific bones. In recent years, generic deep learning-based models have appeared that allow the segmentation of any model through CT images. Regarding the analysis of the fracture zone, several filters stand out in the literature. However, it is the purpose of the study to determine the accuracy and the effort that should be put into this task.

There are four representations of bone fracture patterns in the literature. Texture-based are quite useful when you want to reduce the process to obtain it. The representation based on point clouds, and their variants, is the most used. Its main disadvantages are the time required for its acquisition and the need for the user to intervene during the process. This provides a very faithful representation of the real fracture zone. The contour plot representation has not been widely used in recent years because the information it stores is worse than that stored in point clouds and in the representation based on spherical coordinates. The spherical coordinate representation has been of great value due to the large amount of information it contains. Its use is mainly focused in the line of forensic analysis.

In the future, research could capitalize on the rich information embedded in spherical coordinate representations to advance bone fracture and automatic bone fracture reduction simulations. The detailed fracture characteristics and arrangement along the bone surface afforded by this representation hold promise for designing automatic systems aimed at reducing intervention time and enhancing diagnosis accuracy. Furthermore, continued exploration and refinement of deep learning approaches for fracture zone segmentation could pave the way for more efficient and versatile acquisition methods in the medical domain.

## Figures and Tables

**Table 1:** Summary of the characteristics, advantages, and disadvantages of the segmentation methods found in the literature.

Method	Characteristics	Advantages	Disadvantages
Threshold	- Divide images using the color intensity of the pixels. - Use a threshold to filter bone from other tissues.	- Simplicity. - Effective in situations where the pixel intensities are clear. - Effective segmentation for healthy bones.	- Manual corrections may be necessary. - It has problems in areas where the pixel intensity is not very clear. - Less effective for identifying fractured bones.
Region-growing	- Group pixels or contiguous regions that share similar characteristics. - Some authors combine adaptive thresholding with 2D region growing. - A 3D connected component labeling algorithm can be used to separate the bone fragment. - Several seeds can be used in the same case.	- Effective noise management. - Region-growing approach adapts well to various bone structures. - Effective for segmenting both healthy and fractured bones.	- Complexity increases with multiple fragments. - Adequate sensitivity must be established. - Manual intervention may be needed for accurate segmentation.
Deep learning	- Use neural networks for bone segmentation, achieving high accuracy. - Various strategies employed for model training, including 2D axial slices and pseudo-3D approaches.	- High accuracy in segmentation results. - Less dependent on user input compared to traditional methods. - No manual intervention required.	- Requires large training datasets. - Computational resources may be intensive. - They are sensitive to data quality. - Setting hyperparameters requires prior knowledge.

**Table 2:** Summary of the characteristics, advantages, and disadvantages of the methods used to acquire a bone fracture area representation.

Method	Characteristics	Advantages	Disadvantages
Shape Analysis	- Use cylindrical bone shapes to identify fracture vertices. - Relies on normal orientation comparison with bone axis.	- Effective for cylindrical areas. - Automated vertex identification.	- Ineffective for non-cylindrical bones. - Limited applicability to specific areas of bones.
Interactive Contour Tracing	- Allows specialists to interactively trace fracture contours from segmented bone fragments.	- User involvement facilitates precise fracture identification. - Flexible application across different fracture types.	- Time-consuming due to manual tracing. - Dependent on user expertise.
Statistical Classification	- Utilizes mixture models for binary classification of intact and fractured zones. - Identifies fractured surface as largest continuous region of fracture points.	- Objective classification based on statistical parameters. - Clear delineation of fractured area. - No manual process required.	- Limited to binary classification. - Vulnerable to noise and outliers. - May be inaccurate
Curvature Analysis	- Extracts fracture lines based on curvature analysis of CT slices. - Uses interactive line-tracking software for extraction. - Removes outliers with connectivity test.	- Captures fracture morphology accurately. - Allows for interactive adjustment of fracture lines.	- Manual adjustment may be needed for precise fracture delineation. - Complexity increases with fracture complexity.
Normal Filter	- Identifies candidate points for fracture surface based on normal orientation. - Utilizes connected component analysis for outlier removal.	- Automated detection of fracture candidate points. - Efficient outlier removal with connected component analysis.	- Limited effectiveness for highly complex fractures. - May require manual adjustment for optimal results.
Grid	- Uses an OBB to discretize models and identify fracture candidate points. - Filters candidate points based on distance to opposite fragments.	- Systematic candidate point identification. - Incorporates geometric considerations for fracture reduction.	- Relies on discretization accuracy. - Manual cleaning may still be necessary for precise fracture delineation.
Seed Iterative Filtering	- Uses region growing to study the continuity of the regions that make up a fracture.	- Reduces manual cleaning with automated filtering process. - Iterative refinement enhances accuracy.	- Complexity increases with fracture complexity. - Computational cost may be high. - User intervention is required.
Density Clustering	- Defines clusters based on point cloud density.	- Automated detection of fracture zones.	- Vulnerable to noise and deformations. - Fracture zones may contain high levels of noise.

**Table 3:** Overview of advantages and disadvantages of each bone fracture pattern representation.

Method	Advantages	Disadvantages
Texture	- Simple and easy to obtain and use. - Suitable for combining pattern-based and physics-based approaches to fracture simulation.	- Insufficient representativeness of fracture pattern. - Lack of comprehensive details about the fracture zone from a 3D perspective. - User involvement needed in the acquisition process.
Point Clouds	- Provides a large amount of information. - There are different ways to represent the information. - Widely used representation for human bone simulations. - Used to represent both healthy and fractured zones. - Information can be extrapolated to other clinical cases. - Familiar representation to real clinical cases. - Offers different perspectives for observation.	- Requires complex image analysis techniques for acquisition. - User involvement needed in the acquisition process.
Spherical Coordinates	- Allows for faithful representation of the fracture zone. - Enables detailed analysis of fracture characteristics. - Allows identification of the areas of the bone in which fracture lines occur.	- Complex process required for 3D environment use. - Requires reverse projection for representation. - Dealing with deformations at bone ends can be challenging.
Contour Plots	- Allows users to store the direction in which the fracture occurred. - Allows us to work on fracture reduction in a simple way.	- Complex usage in a 3D environment. - Requires reverse projection for representation. - Dealing with deformations at bone ends can be challenging. - Insufficient representativeness of fracture pattern.

## Data Availability

All data are available by corresponding author upon reasonable request.
